# Oral administration of 6-methylsulfinylhexyl isothiocyanate extracted from wasabi is safe and improves the fatigue and sleep of healthy volunteers

**DOI:** 10.1186/s13030-023-00287-0

**Published:** 2023-08-23

**Authors:** Ryota Nakajima, Masanobu Kanou, Masahiko Tokushima, Yoshitaka Iwama, Kei Yamana

**Affiliations:** 1 Nutraceutical Group, Division of New Business in Healthcare Business, Teijin Ltd., 2-1, Kasumigaseki 3-chome, Chiyoda-ku, Tokyo, 100-8585 Japan; 2 NOMON Co., Ltd, Tokyo, Japan; 3 Maebashi North Hospital, Maebashi, Japan; 4 Nihonbashi Cardiology Clinic, Tokyo, Japan

**Keywords:** Wasabi extract powder, 6-MSITC, Fatigue, Sleep, Safety study, Clinical study

## Abstract

**Background:**

This study aimed to conduct a preliminary evaluation of the effects of 6-methylsulfinylhexyl isothiocyanate (6-MSITC) contained in wasabi rhizomes on fatigue and sleep and to examine its safety through overdose study.

**Methods:**

A total of 20 healthy volunteers who were experiencing daily fatigue were given powder containing 6-MSITC (4.8 mg/day of 6-MSITC) extracted from wasabi for 4 weeks. Then, fatigue, sleep, autonomic nervous functioning, stress, and immunity were evaluated. In addition, an overdose safety study of the extract powder (up to 16 mg/day of 6-MSITC for 4 weeks) was performed with 30 healthy volunteers in a double-blind, placebo-controlled method.

**Results:**

The powder containing 6-MSITC did not improve fatigue after a mental task, but fatigue before the mental task, sleep, and mood were improved significantly after 4 weeks intake. No changes were observed in the autonomic nerve function, stress, or immune markers. In the overdose safety study, no changes in the parameters ​​or side effects were observed, and the results showed that high doses of the extract powder containing 6-MSITC is safe.

**Conclusion:**

This study confirmed the possibility that this powder extracted from wasabi that contains 6-MSITC might improve fatigue and sleep. However, because the effectiveness evaluation in this study was a single-arm, open-label study and there was no placebo control group, these points must be considered when interpreting the results. Safety was confirmed in an overdose study of more than three times the amount compared to that in the efficacy evaluation study. In the future, further research should be conducted on its effectiveness for treating fatigue and sleep problems.

**Trial registration:**

UMIN clinical trial registration system, UMIN000049913. Registered 27 December 2022 Retrospectively registered, https://center6.umin.ac.jp/cgi-open-bin/ctr/ctr_view.cgi?recptno=R000056818

## Background

Wasabi is a plant native to Japan that has a long history of being consumed as a medicinal herb. Bioactive isothiocyanate compounds have been found in wasabi, and 6-methylsulfinylhexyl isothiocyanate (6-MSITC) is the major component [1]. Nonclinical studies have reported that 6-MSITC shows antioxidant [2], anti-inflammatory [3], antibacterial, anticancer, antiplatelet aggregation [4–8], antidiabetic [9], and antiallergic activity [10]. Furthermore, intake of 6-MSITC improved cognitive impairment in mouse models of Alzheimer’s disease [11]. Our in-house study demonstrated that the administration of 1.6 mg of 6-MSITC for 4 weeks decreased the level of oxidative stress in urine and semen [12]. It was also shown to improve cognitive function in healthy volunteers at dose of 0.8 mg for 8 weeks [13]. In addition, studies on patients with myalgic encephalomyelitis/chronic fatigue syndrome have reported that 6-MSITC at dose of 9.6 mg/day for 12 weeks significantly improves the performance status, cognitive dysfunction, and cognitive performance of patients, such as for the Trail Making Test-A and subjective symptoms of brain fog [14]. Based on these findings, powder extracted from wasabi that contains 6-MSITC is attracting attention as a functional food ingredient in the “Food with Function Claims” of maintained and improved cognitive function at doses of 0.8 or 1.6 mg/day.

6-MSITC is known to activate the transcription factor nuclear factor erythroid 2-related factor 2 (Nrf2) [15]. Activated Nrf2 translocates into the nucleus, binds to the antioxidant response element, and enhances the transcription of antioxidant proteins and phase II metabolic enzymes, thereby exerting antioxidant and detoxifying metabolic enzyme-inducing effects. 6-MSITC suppresses lipopolysaccharide-stimulated cyclooxygenase-2 expression, which is closely related to inflammation [3]. Furthermore, 6-MSITC has various biochemical effects, including an anticancer effect exerted by acting on the mitochondria of cancer cells and inducing apoptosis [6].

Due to the wide range of reported physiological activities, as described above, novel potential benefit of 6-MSITC against fatigue were explored in the present study. Given its combined antioxidant and anti-inflammatory properties, we hypothesized that 6-MSITC might show efficacy against fatigue, which is a significant alarm signal in the body triggered to prevent damage caused by excessive exercise. Damage to cells or tissues due to excessive exercise is mainly caused by oxidative stress, and fatigue occurs when there is insufficient energy to repair this cell damage [16, 17]; Inflammation caused by cell damage also induces fatigue. Thus, 6-MSITC is likely to have an anti-fatigue effect and may affect sleep [18–20], stress [20], autonomic nerve function [19], and immunity [18], which are related to fatigue. Therefore, we conducted a preliminary single-group open-label study to broadly understand the effects of 6-MSITC, with the aim of doing a future double-blind placebo-controlled study to clarify the effects of 6-MSITC. In addition, the powder containing 6-MSITC used in the experiments is mainly used as a supplement that contains 0.8% 6-MSITC. The safety of high doses of the ingredient was also evaluated in an overdose study to verify that it can be ingested safely.

## Methods

### Study design

The effectiveness evaluation study was a single-arm, open-label study, whereas the overdose safety study was a placebo-controlled, randomized, double-blind, parallel-group study.

### Test extract

Test capsules with 200 mg of wasabi extract containing 1.6 mg of 6-MSITC and placebo capsules that did not contain the wasabi extract were used. The capsule compositions are summarized in Table 1. The wasabi extract was manufactured by Kinjirushi Co., Ltd.

**Table 1:** Composition of capsules

	Test capsules	Placebo capsules
Ingredients	Wasabi extract powder containing 6-MSITC, α-cyclodextrin, calcium stearate	α-cyclodextrin, calcium stearate
Amount of active ingredients per capsule	Wasabi extract powder containing 6-MSITC 200 mg (contains 1.6 mg of 6-MSITC)	0 mg

6-MSITC: 6-methylsulfinylhexyl isothiocyanate

### Participants

The effectiveness evaluation study complied with the Declaration of Helsinki guidelines and was approved by the Nihonbashi Cardiology Clinic Examination Committee (approval date: December 1, 2021, approval number: NJI-021-12-02), registered in the UMIN clinical trial registration system, published (clinical trial registration number: UMIN000046580), and conducted with sufficient safety considerations. Healthy volunteers were informed about the study content by the principal investigator, and consent was obtained in writing for their voluntary participation.

Healthy volunteers were recruited by KSO Co., Ltd. Among the 43 individuals who agreed to participate, 20 healthy men and women (age 43.4 ± 6.3 years) who met the following selection criteria and did not meet the exclusion criteria were selected as participants. The selection criteria were as follows: (1) Japanese men and women between the ages of 35 and 60 years who experienced fatigue daily and (2) those who received a sufficient explanation of the purpose and contents of the study, had the ability to provide consent, voluntarily applied for participation after having a good understanding of the study, and agreed to participate in the study in writing. The exclusion criteria were as follows: (1) a history of serious liver, renal, or heart disease; (2) suffering from a disease and were receiving outpatient treatment, medication, or treatment; (3) taking medicines, supplements, or health foods related to recovery from fatigue and lack of sleep; (4) suspected in a screening test to have depression, menopausal disorder, or sleep disorder; (5) diagnosed with chronic fatigue syndrome; (6) declared that they were allergic to the ingredients of the test food; (7) taking medicines or supplements that might affect the results of the study; (8) pregnant, breastfeeding, or intending to become pregnant during the study period; (9) irregular shift work, late-night work, etc.; (10) heavy alcohol drinkers (drinking 60 g or more of pure alcohol or an equivalent at least 5 days a week); (11) participating or intending to participate in studies involving ingesting other foods or taking drugs or in studies on applying cosmetics or drugs; and (12) considered to be inappropriate as study participants by the principal investigator. In addition, participants were given the following instructions during the study period: (1) the test extract must be taken as instructed by the research director, (2) the test extract should not be consumed by anyone other than the study participants themselves, (3) do not consume alcohol from 2 days before a test, (4) go to bed early the day before all tests, (5) do not eat or drink anything other than water after 21:00 on the day before the tests, (6) refrain from having dental treatment during the 2 days before the tests so as not to affect the saliva test, (7) do not change eating habits or lifestyle habits significantly during the study period compared to before the study (do not overdose, overeat, diet, travel abroad, abruptly stop exercising, start new exercise, etc.), (8) refrain from taking or ingesting drugs, supplements, health foods (including foods with function claims) related to recovery from fatigue and lack of sleep during the study period, and (9) refrain from participating in studies during the study period involving consumption of other foods, use of drugs, or studies involving the application of cosmetics or pharmaceuticals.

The overdose safety study followed the Declaration of Helsinki and was approved by the Medical Corporation Kimiokai Kobuna Orthopedic Clinic Ethics Review Committee (approval date: August 16, 2018, approval number: MK1808-2), registered in the UMIN clinical trial registration system, published (clinical trial registration number: UMIN000049913), and conducted with sufficient safety considerations. Healthy volunteers were recruited by the KSO Co., Ltd. Among the 75 people who agreed to participate, 30 healthy men and women who met the following selection criteria and did not conflict with the exclusion criteria were selected as participants. Ten participants each were assigned to a placebo-control group, a high-dose group (16 mg/day of 6-MSITC), and a low-dose group (8 mg/day of 6-MSITC). The selection criteria were as follows: (1) healthy men and women at least 20 years old and under 65 years old at the time of providing consent to participate in the study and (2) those who received a sufficient explanation of the purpose and contents of the study, had the ability to provide consent, voluntarily applied for participation after having a good understanding of the study, and agreed to participate in the study in writing.

### Intervention schedule

The effectiveness evaluation study included a 2 week pre-observation period followed by a 4 week intake period, after the participants were screened. During the intake period, the participants took 3 capsules (containing 4.8 mg of 6-MSITC) once a day with water or lukewarm water at approximately 10:00 am (between meals, on an empty stomach). In case of forgetting to take it, they were advised to retake it between meals or on an empty stomach. However, if a participant did not take a dose orally, it was regarded as a missed dose, and they were not allowed to take more than 4 capsules at once the next day. The dose of the effectiveness evaluation study was based on previous studies, we selected a dose of 3 capsules [12–14].

In the overdose safety study, the intake period was 4 weeks, and the post-intake observation period was 2 weeks. The participants took 10 capsules (16 mg/day of 6-MSITC) once a day, divided into several capsules, with water or lukewarm water at approximately 10:00 am (between meals and on an empty stomach). The capsules consisted of 10 placebo capsules for the placebo-control group, five placebo plus five test capsules for the low-dose group, and 10 test capsules for the high-dose group. The dose of the overdose safety study was fixed at 10 times higher than that of the major dose of 6-MSITC contained in the dietary supplement.

### Outcome measures

The effectiveness study preliminarily evaluated the effects of 6-MSITC on fatigue, sleep, autonomic nervous system functioning, stress, and immunity. The primary endpoint was the visual analog scale (VAS) of fatigue, which was performed before and after the mental task. The Uchida-Kraepelin test was performed as a mental task for 60 min at weeks 0 and 4 of the intake periods [21]. The shortened version of POMS II and the autonomic nerve function evaluation were performed as secondary endpoints at the same timing as for the VAS for fatigue. Autonomic nerve function was tested by measuring the fingertip pulse waves for 2 min each with Ultet (CDN type) manufactured by Yumedica Co., Ltd. VAS was performed every week after the start of intake to evaluate the efficacy for sleep. The evaluation items of this VAS consisted of 10 items: “mental fatigue”, “physical fatigue”, “recovery of fatigue”, “refresh”, “ability to concentrate”, “stress”, “lightness of the body”, “ease of falling asleep”, “quality of sleep”, and “sleepiness of rising”. Salivary cortisol and α-amylase were evaluated as stress-related outcomes, and salivary sIgA and blood immunoglobulin G (IgG) were evaluated as immune-related outcomes before and after intake. Approximately 0.8 mL of naturally secreted saliva was collected after participants rested for 5 min and swallowed the saliva in their mouths. In terms of safety assessment, medical interviews, body weight (body mass index, BMI), blood pressure/heart rate, laboratory test (hematology, blood biochemistry, and urinalysis), subjective and objective symptoms, and adverse events were evaluated.

The evaluation items of the overdose safety study included medical interviews, BMI, physiological tests (blood pressure and heart rate), hematological tests (white blood cell count, red blood cell count, hemoglobin, hematocrit, mean corpuscular volume (MCV), mean corpuscular hemoglobin (MCH), mean corpuscular hemoglobin concentration (MCHC), platelet count, and white blood cell images), blood biochemistry tests (total protein, albumin, total bilirubin, aspartate transaminase (AST), alanine transaminase (ALT), lactate dehydrogenase (LD), alkaline phosphatase (ALP), γ-glutamyl transferase (γ-GT), creatine kinase (CK), urea nitrogen, uric acid, creatinine, sodium, chloride, potassium, calcium, inorganic phosphorus, magnesium, serum iron, total cholesterol, low density lipoprotein (LDL)-cholesterol, high density lipoprotein (HDL)-cholesterol, triglyceride (TG), fasting blood glucose, and hemoglobin A1C (HbA1c)), urinalysis (protein, glucose, urobilinogen, bilirubin, pH, specific gravity, ketone body, and occult blood reaction), subjective symptoms, and adverse events conducted at the start of intake (week 0), 2 weeks later (week 2), 4 weeks later (week 4), and after the follow-up period (weed 6).

### Statistical analysis

Statistical analysis of the effectiveness studies was performed using IBM SPSS statistics version 24. The significance level was set at 5%.

The average value and standard deviation were obtained from each group for each data in the statistical analysis of the overdose safety study. Dunnett’s test (two-tailed test) was used to perform statistical analysis of the high-dose group, low-dose group, and control group for each test, and Wilcoxon’s rank-sum test was performed for qualitative endpoints. A stratified analysis was performed separately for men and women for measurement items with different reference values for men and women. Statistical analysis was performed using Dunnett’s test (two-tailed test) for the comparison between the intake start date (week 0) and the week 2, week 4, and follow-up (week 6) tests after the start of intake, whereas Wilcoxon’s signed-rank test was performed for qualitative endpoints. The statistical significance level was set at 5%. IBM SPSS Statistics version 24 was used for data analysis.

## Results

### Efficacy evaluation study

#### Participants

Of the 20 participants in the study, one male participant tested positive for coronavirus disease 2019 (COVID-19) after intake and was unable to participate in any tests other than the VAS assessment of sleep at waking. As a result, only the VAS of sleep at waking was analyzed with all 20 participants. The primary endpoints and other measurement items were assessed with the data of 19 participants. Table 2 summarizes the background of the participants.

**Table 2:** Effectiveness evaluation participant background

Item	
Sex (number)	Male (11), Female (9)
Age	43.4 ± 6.3
Body weight (Kg)	66.4 ± 14.1
BMI	23.8 ± 4.0
Systolic blood pressure (mmHg)	123.8 ± 15.2
Diastolic blood pressure (mmHg)	79.8 ± 10.9
Heart rate(bpm)	71.5 ± 7.6

Mean ± standard deviation, BMI: body mass index

#### Efficacy evaluation

**Fig. 1:**
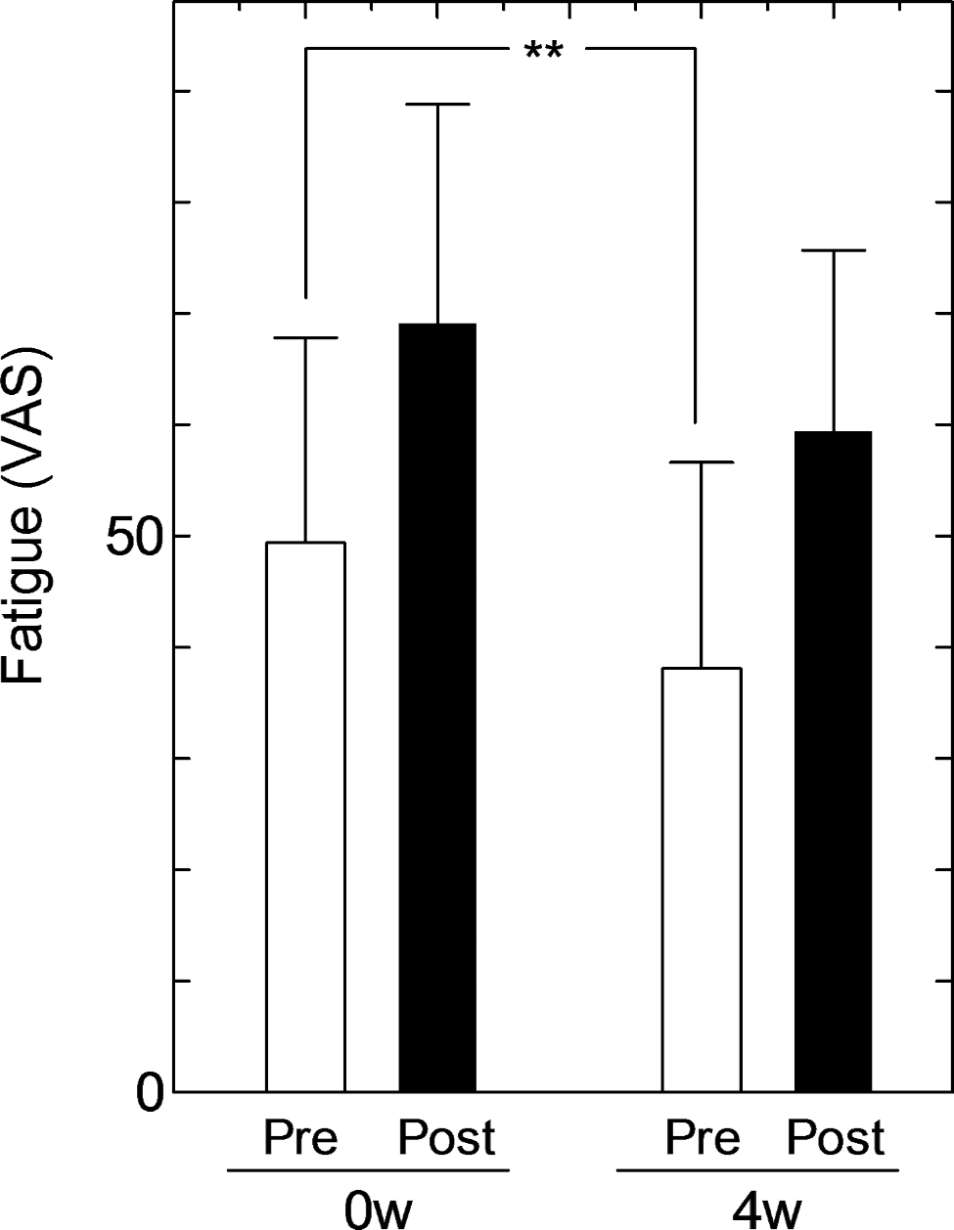
Changes of VAS scores of fatigue after treatment with extract Pre: before the Uchida-Kraepelin test, Post: after the Uchida-Kraepelin test Mean ± standard deviation, n = 19, **p < 0.01

Figure 1 summarizes the results of the VAS of fatigue before and after taking 6-MSITC for 4 weeks. There was no significant change in fatigue or the increase in fatigue due to the mental task between weeks 0 and 4. Fatigue before the mental task was significantly reduced after 4 weeks of intake compared to before intake. The T scores of POMS after the mental task did not change for before and after intake, On the other hand, T scores before the mental task, such as “confusion-bewilderment”, “depression-dejection” “fatigue-inertia”, “tension-anxiety” and “total mood disturbance (TMD)” were significantly improved compared to week 0 (Table 3). Table 4 summarizes the VAS of sleep at waking (The lower the value of VAS in this study, the better the condition). All items except “ease of falling asleep” significantly improved depending on the intake period. In particular, “quality of sleep” improved from 1 week after intake, and “sleepiness of rising” improved from 2 weeks after intake. There were no significant changes in autonomic nerve function, salivary cortisol, salivary α-amylase, or salivary sIgA, but blood IgG significantly decreased (Table 5).

**Table 3:** Changes in POMS (T-score) after treatment

Mood factors		T-score	
		0w	4w
Anger-Hostility	Pre Post Δ	50.7 ± 7.5 47.9 ± 8.6 -2.8 ± 6.8	48.1 ± 9.1 46.5 ± 9.1 -1.5 ± 6.6
Confusion-Bewilderment	Pre Post Δ	49.4 ± 6.8 51.9 ± 8.6 2.5 ± 8.3	44.9 ± 5.3* 49.6 ± 9.0 4.7 ± 9.5
Depression-Dejection	Pre Post Δ	48.4 ± 6.8 45.9 ± 7.8 -2.5 ± 4.4	44.1 ± 3.7** 44.8 ± 5.5 0.8 ± 5.3
Fatigue-Inertia	Pre Post Δ	51.3 ± 7.9 57.1 ± 9.5 5.7 ± 8.4	45.5 ± 6.4** 51.4 ± 9.5* 5.9 ± 10.6
Tension-Anxiety	Pre Post Δ	50.6 ± 9.3 47.5 ± 7.7 -3.1 ± 3.7	43.6 ± 5.0*** 43.7 ± 8.2* 0.1 ± 6.2
Vigor-Activity	Pre Post Δ	52.5 ± 8.3 50.4 ± 10.7 -2.2 ± 8.3	54.2 ± 8.4 49.0 ± 9.8 -5.2 ± 7.3
Friendliness	Pre Post Δ	52.9 ± 9.9 45.6 ± 12.1 -7.3 ± 10.9	51.0 ± 9.6 45.8 ± 12.8 -5.2 ± 9.9
TMD	Pre Post Δ	49.7 ± 6.8 50.3 ± 7.4 0.5 ± 5.1	44.2 ± 5.0** 47.4 ± 7.7 3.2 ± 7.3

Mean ± standard deviation, n = 19.

*p < 0.05, **p < 0.01, ***p < 0.001　vs. 0 weeks.

Pre: before the Uchida-Kraepelin test, Post: after the Uchida-Kraepelin test.

TMD: Total Mood Disturbance.

**Table 4:** Changes of VAS scores of sleep at waking after treatment

Item	VAS score				
	0 w	1 w	2 w	3 w	4 w
Mental fatigue	48.9 ± 19.3	49.3 ± 20.5	46.1 ± 18.8	37.5 ± 20.2*	37.1 ± 20.6*
Physical fatigue	53.6 ± 19.2	47.8 ± 24.1	46.3 ± 16.5	41.6 ± 19.9*	39.9 ± 18.9**
Recovery of fatigue	57.5 ± 22.8	48.6 ± 25.7	49.8 ± 21.8	44.4 ± 22.6	42.4 ± 18.2*
Refresh	51.9 ± 22.9	49.1 ± 19.0	47.5 ± 22.0	39.0 ± 27.0*	39.7 ± 22.5*
Ability to concentrate	52.3 ± 20.5	49.6 ± 18.7	46.9 ± 19.0	38.9 ± 17.8**	39.8 ± 19.8*
Stress	59.2 ± 16.1	53.4 ± 22.4	48.3 ± 22.3	44.2 ± 19.6**	40.7 ± 20.0***
Lightness of the body	56.8 ± 17.9	48.8 ± 20.1	50.9 ± 19.5	42.6 ± 22.1*	41.7 ± 20.9**
Ease of falling asleep	42.1 ± 24.5	38.2 ± 20.8	41.8 ± 23.7	33.1 ± 19.1	35.1 ± 16.6
Quality of sleep	53.0 ± 23.7	40.6 ± 19.2*	37.2 ± 23.3**	34.4 ± 20.5**	36.2 ± 17.5**
Sleepiness of rising	55.4 ± 22.3	44.0 ± 20.8	41.1 ± 19.9*	35.1 ± 20.3***	35.7 ± 22.8**

Mean ± standard deviation, n = 20.

*p < 0.05, **p < 0.01, ***p < 0.001 vs. 0 weeks.

**Table 5:** Changes of Autonomic function, stress, and immunity after treatment

Item	Load	Measured values	
		0 w	4 w
LF/HF-MEM	Pre Post	2.1 ± 2.0 2.7 ± 3.2	2.1 ± 2.1 2.8 ± 2.6
LF/HF-FFT	Pre Post	1.8 ± 1.5 2.2 ± 2.7	1.9 ± 1.8 2.6 ± 2.7
Salivary cortisol(mg/dL)	-	0.16 ± 0.07	0.15 ± 0.07
Salivary amylase(U/mL)	-	59.8 ± 30.0	63.4 ± 33.2
Salivary sIgA(mg/mL)	-	140.6 ± 55.3	129.8 ± 47.1
Blood IgG(mg/dL)	-	1182 ± 200	1156 ± 186*

Mean ± standard deviation, n = 19.

*p < 0.05 vs. 0 weeks.

Pre: before the Uchida-Kraepelin test, Post: after the Uchida-Kraepelin test.

LF: power in low frequency range, HF: power in high frequency range, MEM: maximum entropy method, FFT: fast Fourier transform, sIgA; secretary immunoglobulin A, IgG; immunoglobulin G.

#### Safety

In this study, there were no significant adverse events, medical interventions, subjective or objective symptoms, or changes in BMI. There were five adverse events in 4 out of 20 cases during the study period. However, the investigator judged that there was “no causal relationship” between the adverse event and the test extract (Table 6).

**Table 6:** Observed adverse events in effectiveness evaluation study

Number of cases	Adverse event	Time of onset	Severity	Procedures	Causal relationship with test extract
2	Shoulder stiffness	Intake period	Mild	None	None
1	Pain of left-hand thumb	Intake period	Mild	None	None
1	Pain of left upper arm, neck and shoulder	Intake period	Moderate	None	None
1	COVID-19	Intake period	Moderate	None	None

### Overdose safety study

#### Participants

All 30 participants in the study were included in the analysis because there were no discontinuations. The background of participants is summarized in Table 7.

**Table 7:** Overdose safety test participant background

Item	Group		
	Control	Low-dose	High-dose
Sex(number)	Male (5), Female (5)	Male (4), Female (6)	Male (5), Female (5)
Age	41.3 ± 14.9	41.2 ± 15.7	41.8 ± 11.1
Height (cm)	164.3 ± 6.6	164.4 ± 10.7	163.9 ± 7.8
Body weight (Kg)	65.3 ± 7.7	61.0 ± 10.4	60.5 ± 10.3
BMI	24.3 ± 3.2	22.4 ± 1.6	22.5 ± 2.9
Systolic blood pressure (mmHg)	118.6 ± 12.4	118.5 ± 9.9	112.0 ± 9.9
Diastolic blood pressure (mmHg)	73.1 ± 6.7	72.4 ± 9.1	70.1 ± 5.6
Heart rate (bpm)	70.2 ± 8.4	68.4 ± 7.9	65.3 ± 8.2

Mean ± standard deviation, BMI: body mass index

#### Safety evaluation

There were no problematic findings in the medical interviews and no serious adverse events during the study. There were eight adverse events observed during the study period (Table 8), all adverse events were judged by the investigator to be “unrelated” to the test extract, and there were no side effects. The physiological tests did not show any significant variation in measurements. Hematological tests showed significant changes in MCH, neutrophils/white blood cell images, lymphocytes/white blood cell images, and monocytes/white blood cell images; however, the investigator judged that there were no safety problems because all values were within the reference values and within the range of physiological fluctuations. Table 9 summarizes the items for which significant changes were observed in the blood biochemistry tests; the investigator judged that there was no problem in terms of safety because all values were within the reference values and within the range of physiological fluctuations. No significant changes were observed for body weight or urinalysis results.

**Table 8:** Observed adverse events in overdose safety study

Group	Number of cases	Adverse event	Time of onset	Severity	Procedures	Causal relationship with test extract
Control	1	Elevated white blood cell count	Observation period	Mild	None	None
Low-dose	1	Cold	Intake period	Mild	None	None
	1	Protein in urine	Intake period	Mild	None	None
	1	AST and ALT elevation	Observation period	Mild	None	None
	1	Protein in urine	Observation period	Mild	None	None
High-dose	1	CK elevation	Intake period	Mild	None	None
	1	Slight cold	Intake period	Mild	None	None
	1	Ketone bodies in urine	Observation period	Mild	None	None

AST: aspartate transaminase, ALT: alanine transaminase, CK: creatine kinase

**Table 9:**
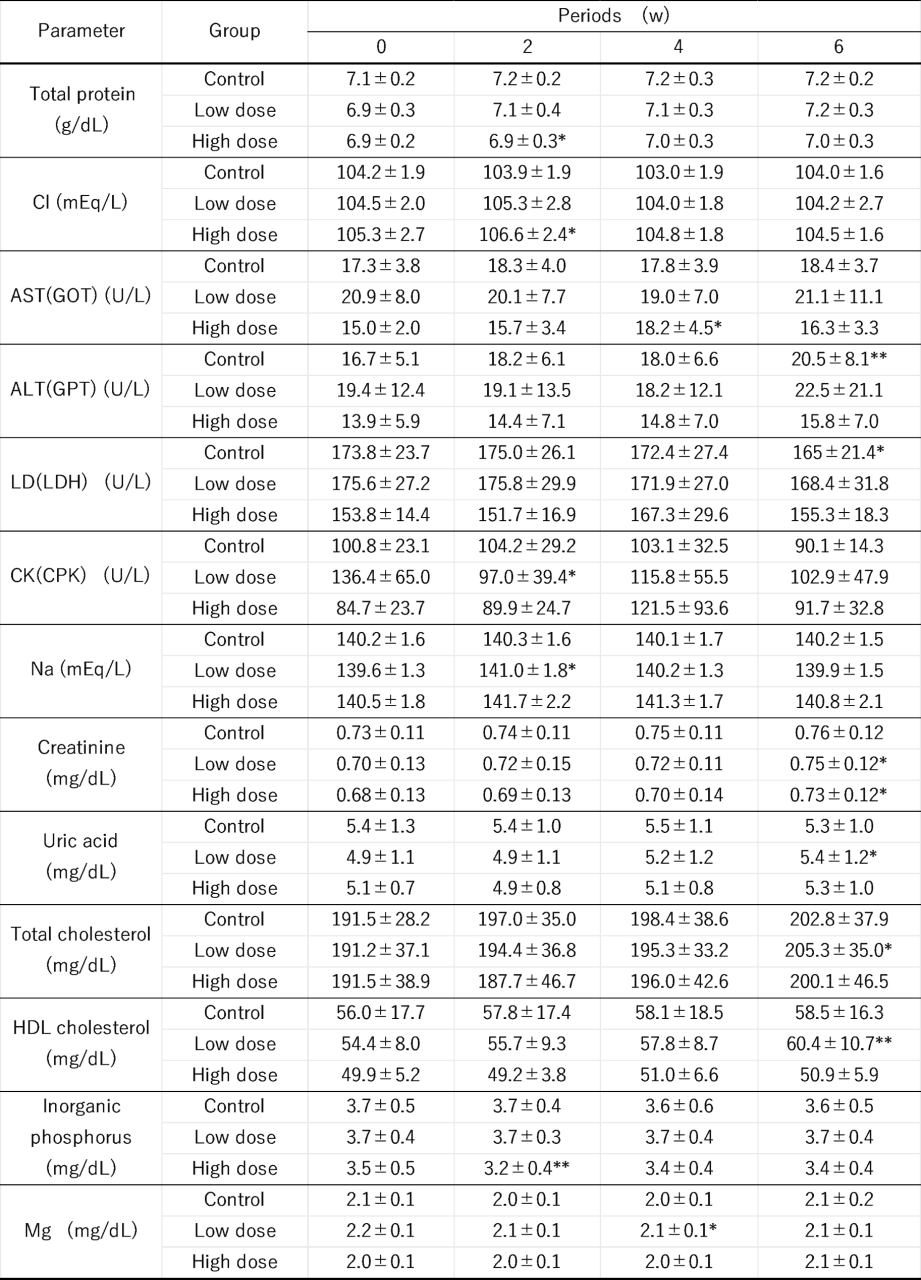
Changes of blood biochemistry test in overdose safety study Mean ± standard deviation

## Discussion

6-MSITC derived from wasabi has been found to have various physiological activities, including strong antioxidant effects in non-clinical studies. In human clinical studies, it has been shown to suppress oxidative stress in urine and semen [12] and to improve cognitive function [13]. Therefore, we conducted a single-group, open-label study with the aim of clarifying the physiological effects of this powder containing 6-MSITC extracted from wasabi. It should be noted that open-label study has a placebo effect.

In this study, we focused on the anti-fatigue effects of 6-MSITC and obtained a wide range of data including sleep, stress, and immunity to evaluate the possibility of 6-MSITC efficacy to inform further double-blind testing. Fatigue includes muscle fatigue caused by exercise and mental fatigue caused by brain activity. Because fatigue due to desk work is a relevant concern in modern society, the Uchida-Kraepelin test, which is a simple calculation task, was extended from the usual 30 min to 60 min [21] to allow the effect of the extract containing 6-MSITC to be evaluated under conditions of a greater mental burden on the brain. The results showed that feelings of fatigue and POMS after the mental burden did not change when taking the test substance for 4 weeks. (Fig. 1; Table 3). However, as shown in Fig. 1, pre-task fatigue was significantly reduced by the intake of 6-MSITC. Similar results were observed with POMS. No change was observed in T-scores after the mental task due to the intake of the test substance; however, for the T-scores before the mental task, significant improvements were observed in “confusion”, “depression”, “fatigue”, “tension” and “TMD” before and after intake. For “fatigue” and “tension”, the scores after the task improved significantly; however, because no changes were observed between before and after the task, we assume that the difference before the task remained the same. These results suggest that the tested extract might be effective not only for fatigue but also on the mood scales. In addition, in the VAS score related to sleep measured on awakening, a significant improvement was observed in the nine items other than “ease of falling asleep,“ depending on the intake period. The score of the participant who tested positive for COVID-19 during the study period was included as it was within the dispersion of values for other participants and was not clearly affected by the infection. These results suggest that long-term intake of 6-MSITC might improve sleep. Therefore, while this study did not observe anti-fatigue effects after the mental task following the administration of 6-MSITC, the results suggest that 6-MSITC might improve daily fatigue and sleep. These results will serve as the basis for a future double-blind study.

Conversely, no significant changes were observed in autonomic nerve function, stress markers, or immune markers, except blood IgG (Table 5). The change was approximately 2% for IgG; although significant, it was not a physiologically meaningful change. However, the markers of the participants of this study were all within the normal range for healthy people; hence, they cannot be used to estimate the effects of the test extract. The participant selection criteria should be reconsidered if the effects on these markers are to be re-evaluated.

Antioxidant and mitochondrial activating effects have been reported as mechanisms of supplemental ingredients that exhibit anti-fatigue effects [22, 23]. Supplemental ingredients with sleep-improving effects involve mechanisms such as stimulation of the parasympathetic nervous system [24], effects on GABAergic neurons [25], antioxidant effects [26], anti-inflammatory effects [27] and a lowered body temperature due to vasodilatory action [28]. Because 6-MSITC has been confirmed to have antioxidant, anti-inflammatory, and physiological effects, it may also affect fatigue and sleep.

The safety of a powdered extract containing 6-MSITC at 4 mg/day has been reported in humans [29]. In this study, we conducted a placebo-controlled, randomized, double-blind, parallel-group comparative study on the overdose safety of two doses of 6-MSITC of 8 mg/day and 16 mg/day. The results did not show any serious adverse events, and no concerning changes were observed in the hematological or blood biochemistry tests.

## Conclusion

This preliminary evaluation of the effects of a powder containing 6-MSITC extracted from wasabi suggests that it might improve sleep and reduce daily fatigue. Because these results were obtained from a single-arm, open-label study, they must be interpreted carefully, and further clinical study will need to be conducted in the future. In addition, a placebo-controlled, randomized, double-blind, parallel-group comparison was conducted in an overdose study involving doses at least three times higher than in the effectiveness evaluation study, and the safety of 6-MSITC even at high doses was confirmed.

## Data Availability

Data sharing is not applicable.

## List of abbreviations

γ-GT: γ-glutamyl transferase
6-MSITC: 6-methylsulfinylhexyl isothiocyanate
ALP: Alkaline phosphatase
ALT: Alanine transaminase
AST: Aspartate transaminase
CK: Creatine kinase
HbA1c: Hemoglobin A1C
HDL: High density lipoprotein
IgG: Immunoglobulin G
LD: Lactate dehydrogenase
LDL: Low density lipoprotein
MCH: Mean corpuscular hemoglobin
MCHC: Mean corpuscular hemoglobin concentration
MCV: Mean corpuscular volume
Nrf2: Nuclear factor erythroid 2-related factor 2
TG: Triglyceride
TMD: Total mood disturbance
